# Improving sentence reading performance in Chinese children with developmental dyslexia by training based on visual attention span

**DOI:** 10.1038/s41598-019-55624-7

**Published:** 2019-12-12

**Authors:** Jing Zhao, Hanlong Liu, Jiaxiao Li, Haixia Sun, Zhanhong Liu, Jing Gao, Yuan Liu, Chen Huang

**Affiliations:** 1 Key Laboratory of Learning and Cognition, School of Psychology, Capital Normal University, Beijing, China; 2 Yang Zhen Central Primary School, Beijing, China

**Keywords:** Dyslexia, Human behaviour

## Abstract

Deficits in the visual attention span (VAS) are thought to hamper reading performance in dyslexic individuals. However, the causal relationship between VAS deficits and reading disability remains unclear. The present study attempts to address this issue by using a VAS-based intervention to explore the possible influence of VAS on reading processes in Chinese children with dyslexia. Given the influence of the heterogeneity of dyslexia on intervention effects, VAS-impaired dyslexic and VAS-intact dyslexic individuals were separately trained. Therefore, there were five groups of participants in this study, including 10 trained dyslexic individuals with VAS deficits and 10 untrained dyslexic individuals with VAS dysfunction as the baseline reference, 10 trained and 10 untrained dyslexic individuals with an intact VAS, and fourteen age-matched normal readers for reference of normal level. All participants completed reading measures and a visual 1-back task, reflecting VAS capacity with non-verbal stimuli and non-verbal responses, before and after VAS-based training. VAS-based training tasks included a length estimation task regarding the bottom-up attention, visual search and digit cancelling tasks targeting top-down attentional modulation, and visual tracking tasks to train eye-movement control. The results showed that visual training only helped improve VAS skills in VAS-impaired dyslexic individuals receiving training. Meanwhile, their silent sentence reading accuracy improved after training, and there was a significant relationship between training improvements in VAS function and reading performance. The current findings suggest that VAS-based training has a far-transfer effect on linguistic level (i.e., fluent reading). These findings suggest the possibility that VAS-related training may help children with dyslexia improve their reading skills.

## Introduction

Developmental dyslexia (DD) is one of the learning difficulties, especially in reading failure in accuracy, fluency and understanding that cannot be explained by problems in educational opportunity, general intelligence, general motivation, or sensory sensitivity^1^. DD has been found to be caused by a multifaceted disorder^2^ including linguistic-level problems (e.g., orthographic deficits^3–5^) and dysfunction in basic cognitive processing such as visual perceptual analysis^6^ and visual spatial attention^7–14^. In particular, a specific deficit has been consistently observed in tasks that have a common aspect, that is, the paralleled processing of multiple visual elements (i.e., simultaneous visual processing)^15–19^. Multiple-element processing ability could be reflected by the visual attention span (VAS). Since reading partially begins with visual decoding that needs to process multi-letter/character strings, the VAS capacity of dyslexic readers has attracted increasing attention in recent years. Bosse *et al*.^15^ proposed the VAS deficit theory of dyslexia and indicated that VAS impairment might independently account for reading difficulties in dyslexic individuals to some extent. A number of studies using group comparisons between dyslexic readers and chronological-age-matched normal readers reported VAS impairments in dyslexic individuals^19–23^. However, it is not clear whether a VAS deficit is one of the critical causes of dyslexia or if it results from reading disabilities^24,25^. The present study attempts to address this question by utilizing a training method focused on VAS-related skills to explore the possible causality between VAS dysfunction and poor reading outcomes.

## Visual Attention Span and its Possible Role in Reading

Whole-/partial-report tasks with 5-letter strings as stimuli were typically utilized to measure VAS ability and to explore the VAS-reading relationship^15^. In whole-report paradigms, participants are asked to orally report as many letters as possible regardless of the order after a five-letter string was briefly presented, and the number of correctly-reported string and the number of correctly-reported letters across all the trials were regarded as the test scores. In partial-report paradigms, the task requires the participants to orally report a particular letter within a string which appeared at a cued position, and the test scores were the number of correctly-reported letters^15^. The higher the score is, the better the visual attention span ability is. Later, position-based analyses on performance in VAS tasks were conducted to demonstrate the distribution pattern of visual attention span, and relevant findings revealed that visual attentional distribution might be modulated by age-related changes and reading experience^26–28^. For beginning readers or children with reading difficulty, the inter-position variability is remarkably able to show differences in identification sensitivity across positions^27,28^; the inter-position difference tends to decrease with increasing age and with the enhancement of reading ability, revealing an enlargement in the window size of the VAS^27^.

Given that reading involves attentional allocation across letters, the number of stimuli that can be simultaneously processed (i.e., a reflection of the VAS capacity) would exert an influence on reading efficiency. The VAS deficit hypothesis^15^ demonstrates a relationship between VAS disorders and poor reading outcomes, based on the connectionist multi-trace memory model of polysyllabic word reading^29^. The model indicated that there were two reading procedures (i.e., global and analytical reading modes) differing in the window size of visual attention from which the orthographic information of text input was taken. In global reading, the visual attention window contains the entire string, and then, the global phonological output is formed; in analytical reading, the window size of visual attention shrinks to serially process a portion of the visual word, and the phonological outputs thus correspond to the relevant parts within the string. Consequently, it could be proposed that the global reading mode requires a larger VAS than the analytical reading mode and that VAS dysfunction mainly restricts the number of parallel-processed letters during reading, which will further affect the global representation of orthographic and phonological information of the sequence^15^.

## Visual Attention Span Capacity of Dyslexic Individuals in Studies of Alphabetic Languages

In the context of alphabetic languages, Valdois *et al*.^30^ first explored VAS capacity in two French children with dyslexia via whole- and partial-report tasks in which the target stimuli were five-letter strings with no meaning. Their findings revealed that one of the two dyslexic children exhibited poorer performance than chronological-age-matched controls did in both whole- and partial-report tasks, suggesting a possible VAS deficit in the dyslexic children. The following studies replicated this VAS impairment with a larger sample of dyslexic participants^15,16,19,21,23,31^. Relevant research found lower scores in whole-/partial-report tasks with 5-letter strings as stimuli among French, English and Portuguese children with dyslexia compared to their chronological age-matched controls^15,16,19,21,23,31^. Furthermore, position-based analysis indicated that the dyslexic individuals exhibited an abnormal pattern of visual attentional distribution in the VAS task^27^. However, most VAS-related tasks use letters as target stimuli, which has been suggested to involve language-related skills such as visual-to-verbal transfer; thus, the poor performance in dyslexic individuals in these visual tasks may reveal impaired symbol-sound mapping rather than a VAS deficit^32,33^. Lobier *et al*.^17^ designed a visual categorization task on the basis of a partial-report task with symbols as non-verbal stimuli and with non-verbal responses to purely examine the VAS-related processing. Their research excluded the possible influence of language processing and reported that French dyslexic adults responded less accurately than age-matched controls, revealing a pure VAS impairment^17,34^. A VAS intervention study on a French girl with dyslexia reported that VAS-based training could normalize her visual attentional distribution pattern in the VAS task, promote efficiency in word identification and improve text reading performance^27^. The training consisted of VAS tasks differing in difficulty levels, as well as tasks of visual search, discrimination, visual matching and visual parsing, which were all suggested to require simultaneous multi-element processing. Similarly, an intervention study^35^ on Italian dyslexic children found that training multi-letter processing (i.e., a whole-report task) could improve participants’ accuracy and speed during word reading, revealing the benefit of VAS training on reading performance. Currently, Zoubrinetzky *et al*.^36^ designed the MAEVA programme targeting the VAS to train a group of French children with dyslexia and found training effects in both of VAS skills and word reading performance. These findings suggest a possible causal link between VAS deficit and reading disabilities.

However, other studies did not report a significant relationship between VAS deficits and reading difficulty. With whole-/partial-report tasks and visual 1-back tasks to measure VAS capacity, it has been reported that 9-year-old German children and Hebrew adults with dyslexia did not differ from non-impaired readers in their VAS-related measurements, and further position-based analyses showed that the patterns of the visual attention window were similar between dyslexic and normal readers^37–39^. The conflicting findings might be attributed, to some extent, to the heterogeneity of dyslexia. It has been reported that approximately 20–30% of children with dyslexia exhibit VAS impairment in the context of alphabetic languages^18,19^, especially individuals with surface dyslexia^40^. Thus, if pseudoword reading and other reading-related measurements substantially relying on phoneme identification and operation were used as the main screening criteria for dyslexia, it is probable that dyslexic individuals with phonological awareness deficits were selected. It has been reported that the prevalence of dyslexic individuals with a pure VAS deficit is approximately 34%, and 17% of dyslexic individuals showed a double disorder characterized by comorbid phonological deficits and VAS deficits^18^. As suggested by the connectionist multi-trace memory model^29^, some researchers indicated that VAS deficits in dyslexic individuals are independent of their phonological problems, in which the former corresponds to the global reading mode and the latter corresponds to the analytical reading mode. Accordingly, it could be proposed that if an individual with dyslexia exhibits an impairment in phonological processing (especially phonemic awareness), then this impairment would cause an obvious problem in letter-by-letter spelling through the sublexical route (i.e., analytical reading mode). Then, the occurrence probability of VAS dysfunction in this individual would be lower than that in dyslexic individuals without phonological deficits; that is, the existence of phonological problems may further decrease the possibility of the presence of VAS deficits in dyslexia. Therefore, future studies should take into account the subtypes of dyslexia.

Moreover, the orthographic transparency of the native language may potentially modulate the VAS-reading relationship^41^. The orthographic depth of the native language may affect the pattern of the reading mode for beginning and developing readers. In languages with shallow orthographies, readers in early developmental stages maps small orthographic units onto relevant phonemes via the rules of grapheme-to-phoneme correspondence (GPC). By contrast, larger orthographic units are more phonologically regular than smaller ones in languages with deep orthographies^42^. It could be proposed that the VAS may exert a more notable influence on the coarse-grain strategy through the globally lexical route than on the small-grain strategy through the sub-lexical route and that reading in languages with deep orthographies may require a wider allocation of VAS resources than reading in languages with shallow orthographies^43^. Findings from cross-language research provided some evidences for the abovementioned inference. Lallier *et al*.^26^ found that French-Basque bilingual children performed better in VAS-related tasks than Spanish-Basque bilingual children, in which French belongs to a language with deep orthography while both of Basque and Spanish are languages with shallow orthographies. Moreover, a study of cross-language comparison reported that only the VAS skills of French adults were related to their scores on an oral reading fluency test, and there were no significant correlations between VAS and fluent reading in either Arabic or Spanish adults^41^, where French has deeper orthographic depth than Spanish and Arabic. Additionally, an intervention study on a French-Spanish bilingual girl indicated that VAS-based training could contribute more greatly to French reading than Spanish reading^32^. The above findings support that the VAS may primarily affect fluent reading in languages with deep orthographies. Consequently, a VAS deficit supposedly causes problems at the level of the global reading mode^30^, and the VAS impairment in dyslexic readers might be more discernible in languages with deep orthographies.

## Visual Attention Span Capacity of Dyslexic Individuals in Studies of Chinese

In contrast to alphabetic languages, Chinese has a particularly deep orthography without strict GPC rules. As a logographic writing system, Chinese characters have complex visual features. The global processing of characters’ visual forms is critical for Chinese reading because it is conducive to establishing the connection between Chinese orthography and semantics and phonology through a whole-lexical route^44^. It has been shown that VAS significantly contributes to Chinese sentence reading in skilled readers, revealing a close relationship between the VAS and Chinese reading^45^. Thus far, relevant reports about the relationship between the VAS and Chinese reading disability have been scarce and inconsistent. Mi^46^ used whole-/partial-report tasks with high-frequency Chinese characters as stimuli to measure the VAS skills. The results showed that the performance of Chinese dyslexic children in these VAS tasks did not differ from that of the age-matched normal readers. Given that the task, with a meaningless character sequence, might be similar to the character-list reading task, participants involuntarily utilized a character-by-character reading procedure in the analytical reading mode rather than regarding the character sequence as an entire representation. In a recent study^22^, researchers adopted a partial-report task with symbols as stimuli and non-verbal response to measure VAS capacity and observed a developmental increase in VAS deficit in Chinese dyslexic children from different primary school grades. The authors explored VAS skills among Chinese dyslexic children from low, middle, and high grades of primary schools, and the results showed that only the dyslexic children in high grades exhibited lower accuracy in the VAS task as compared to the normal readers^22^. Since both the abovementioned studies utilized an age-matched design, which cannot establish a causal link between VAS deficits and dyslexia, Chen *et al*.^20^ adopted a reading level-matched design to further explore whether VAS impairment in dyslexic readers was the result of their reduced reading experience. Their findings showed that dyslexic children performed worse than both the age-matched and reading level-matched controls in the VAS task, revealing that VAS deficits in Chinese dyslexic children cannot be explained only by poor reading abilities^20^. However, the visual stimuli in this study included Chinese characters, radicals, and digits that belonged to verbal materials. Processing these verbal stimuli would involve visual-to-phonological mapping, and individuals with dyslexia showed impairments in this ability. Accordingly, the poor performance in the abovementioned VAS task probably reflected problems in visual-to-verbal transfer rather than dysfunction in visual attentional processing. Moreover, this study only excluded the possibility that VAS dysfunction was the result of a poor reading outcome, but it remains unknown whether the VAS impairment was one of the causes of dyslexia. Therefore, future studies are required to examine whether a pure VAS deficit could (partially) account for reading difficulties in Chinese individuals with dyslexia.

## Aims of the Present Study

A number of studies have explored the VAS capacity in dyslexic readers by comparing them with chronological age-matched controls; however, there were conflicting findings and has been debated surrounding the causal relationship between VAS disorders and reading disabilities, which might be associated with differences in the orthographic depth of the native language and with stimuli properties and task design. Based on relevant studies with alphabetic languages, it could be inferred that the VAS might have a significant relationship with the global reading mode in the context of languages with deep orthographies, in which group differences in VAS capacities between dyslexic and normal readers would be remarkable. However, the use of letter stimuli and oral reports in most previous studies has been taken as evidence against the VAS accounting for poor performance in dyslexic individuals. Therefore, *the first aim of the present study* was to investigate whether there is a causal link between the VAS and reading-related skills in dyslexic children reading Chinese (i.e., a logographic language with a deep orthography) using a VAS-based intervention method to disentangle cause from effect in dyslexia. VAS skills were measured by a parallel visual processing task with non-verbal stimuli and non-verbal report (i.e., visual 1-back task) to reflect pure VAS capacity. Moreover, we divided the trained dyslexic individuals into two groups, VAS-impaired and VAS-intact individuals, and *the second aim of the present study* was to compare the intervention effect between these two groups and examine the possible influence of the heterogeneity of DD on the intervention effect. The two groups of dyslexic individuals received 10 training sessions focusing on VAS-related subcomponents regarding bottom-up stimulus-driven attention abilities and top-down controlled attention. The VAS and reading-related skills were tested before and after VAS-based training. In addition to the two trained groups, there were three control groups: nontrained dyslexic individuals with VAS deficits, nontrained dyslexic individuals with normal VAS function and age-matched normal readers with normal VAS function that were separately regarded as references for baseline and normal reading levels. By comparing differences in the training effects across groups, we expected to further uncover the possible influence of VAS dysfunction on Chinese reading disability and to examine the applicability of the visual attention span deficit hypothesis in Chinese, with its nonalphabetic writing system and particularly deep orthography. According to the relevant literature^20,22^, it was predicted that training focusing on VAS-related skills would contribute to improving VAS capacity and reading skills, particularly among dyslexic children with a VAS deficit.

## Results

The present study first aimed to explore the possible causal link between VAS deficits and reading disability. To do this, we focused on examining whether there were significant improvements in both VAS capacity and reading skills after VAS-impaired dyslexic individuals received VAS-related training; in addition, we also investigated the correlation between the possible training effects on VAS ability and reading performance. Moreover, we included other control groups to better understand the training improvements in trained VAS-impaired individuals with DD. In detail, untrained VAS-impaired dyslexic individuals were recruited as a baseline reference group to exclude the possible influence of changes related to natural growth, and age-matched normal readers were recruited as a normal control reference group to examine whether VAS ability in the trained VAS-impaired individuals with DD approached or reached normal levels after the intervention. First, we conducted one-sample Kolmogorov-Smirnov tests to examine whether the d-prime values in the visual 1-back task, reading performance in each group and each time point showed normal distributions. The results showed significant effects (*p*s < 0.05) in some conditions (e.g., normal readers’ d-prime values and silent sentence reading speed in the pre-test, and the oral (silent) single-character reading speed of the untrained VAS-impaired individuals with DD), revealing that variables in some conditions exhibited skewed distributions. Moreover, our sample size was small. Accordingly, we used the non-parametric Wilcoxon signed-rank test to address the ability changes before and after the intervention within one group and the non-parametric Mann-Whitney U test to compare the performances between groups. To explore the relationship between improvements in the VAS and reading skills, Spearman correlation analyses were further adopted. For the performance in the visual 1-back task, we used accuracy instead of reaction time as the final dependent variable, which was consistent with previous literature^22^, because the response time in the visual 1-back task might include processes other than VAS skills, such as decision-making, selection of different keys, and motor action related to pressing the corresponding keys. Moreover, d-prime values were computed based on accuracy in each position of a string to further analyse VAS improvement from the position-based aspect. In line with previous studies^47^, the speed of single-character reading, judgement accuracy and reading speed in the sentence task were separately regarded as the dependent variables of fluent reading performance at the single-character and sentence levels.

Additionally, to examine the possible influence of the heterogeneity of DD on the intervention effect (i.e., the second aim of the present study), we recruited the trained and untrained groups of VAS-normal individuals with DD and ran similar tests to those previously mentioned to analyse their performance regarding VAS and reading tests.

## Training Effects of VAS and Reading Skills in VAS-impaired Individuals with DD

**Figure 1:**
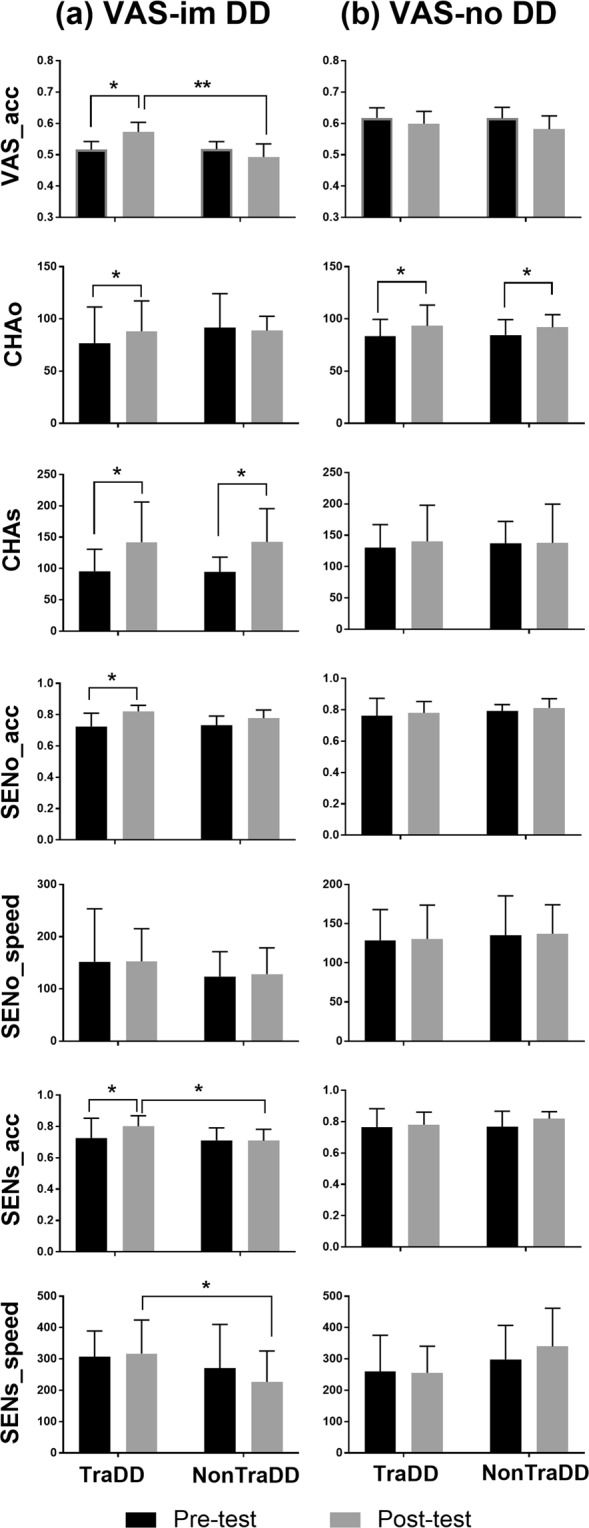
The performance in the visual 1-back task and reading test in pre- and post-tests for the four groups of dyslexic children. (**a**), training effects in VAS and reading skills among trained and untrained dyslexics with VAS deficit; (**b**) training effects in VAS and reading skills among trained and untrained dyslexics with intact VAS function. VAS_acc, mean accuracy in the visual 1-back task; CHAo, oral character reading speed; CHAs, silent character reading speed; SENo_acc, oral sentence reading accuracy; SENo_speed, oral sentence reading speed; SENs_acc, silent sentence reading accuracy; SENs_speed, silent sentence reading speed. VAS-im DD, dyslexics with VAS deficit; VAS-no DD, dyslexics with normal VAS function. TraDD, trained dyslexics; NonTraDD, nontrained dyslexics. Bars in black represent performance before training; bars in grey represent performance after training. Error bars indicate SEM. *p < 0.05; **p < 0.01.

**Figure 2:**
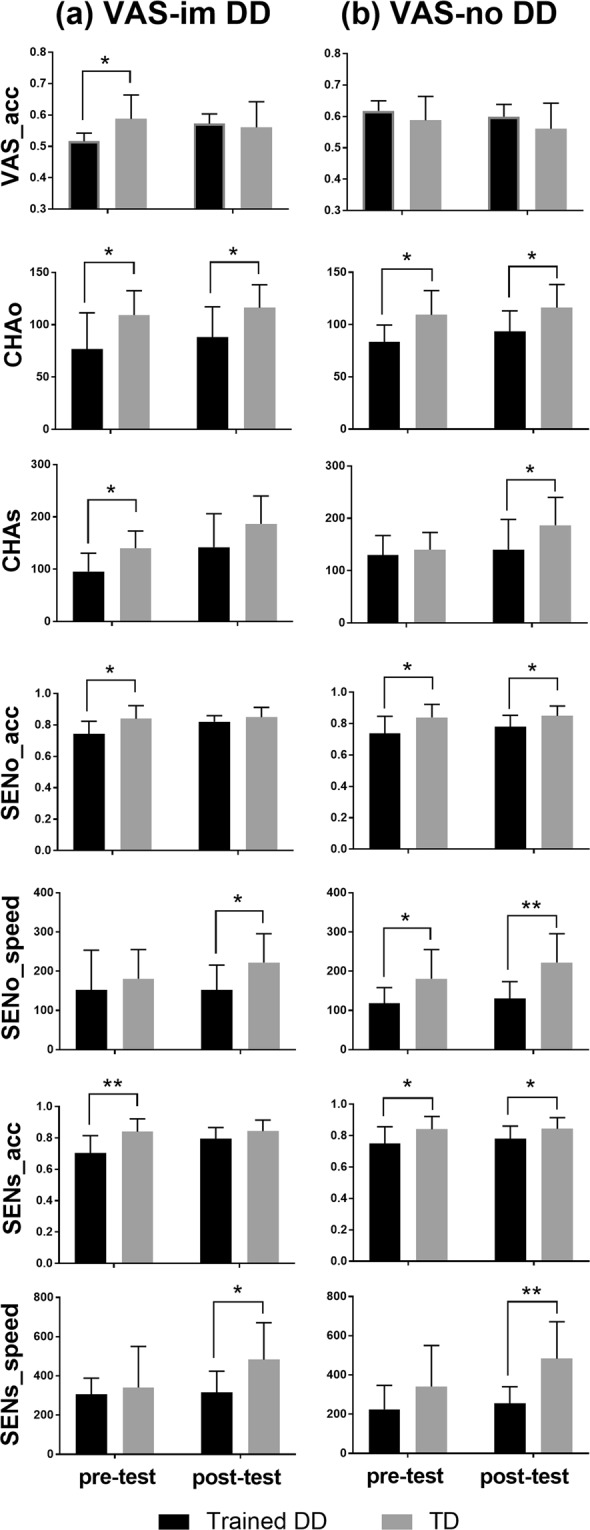
Comparisons in the performance in the visual 1-back task and reading test between trained dyslexics and age matched normal readers in pre- and post- tests. (**a**), comparison between trained dyslexics with VAS deficit and controls; (**b**), comparison between trained dyslexics with normal VAS function and controls. VAS_acc, mean accuracy in the visual 1-back task; CHAo, oral character reading speed; CHAs, silent character reading speed; SENo_acc, oral sentence reading accuracy; SENo_speed, oral sentence reading speed; SENs_acc, silent sentence reading accuracy; SENs_speed, silent sentence reading speed. VAS-im DD, dyslexics with VAS deficit; VAS-no DD, dyslexics with normal VAS function. TD, typically developing children. Bars in black represent performance of trained dyslexics; bars in grey represent performance of age-matched normal readers. Error bars indicate SEM. *p < 0.05; **p < 0.01.

**Figure 3:**
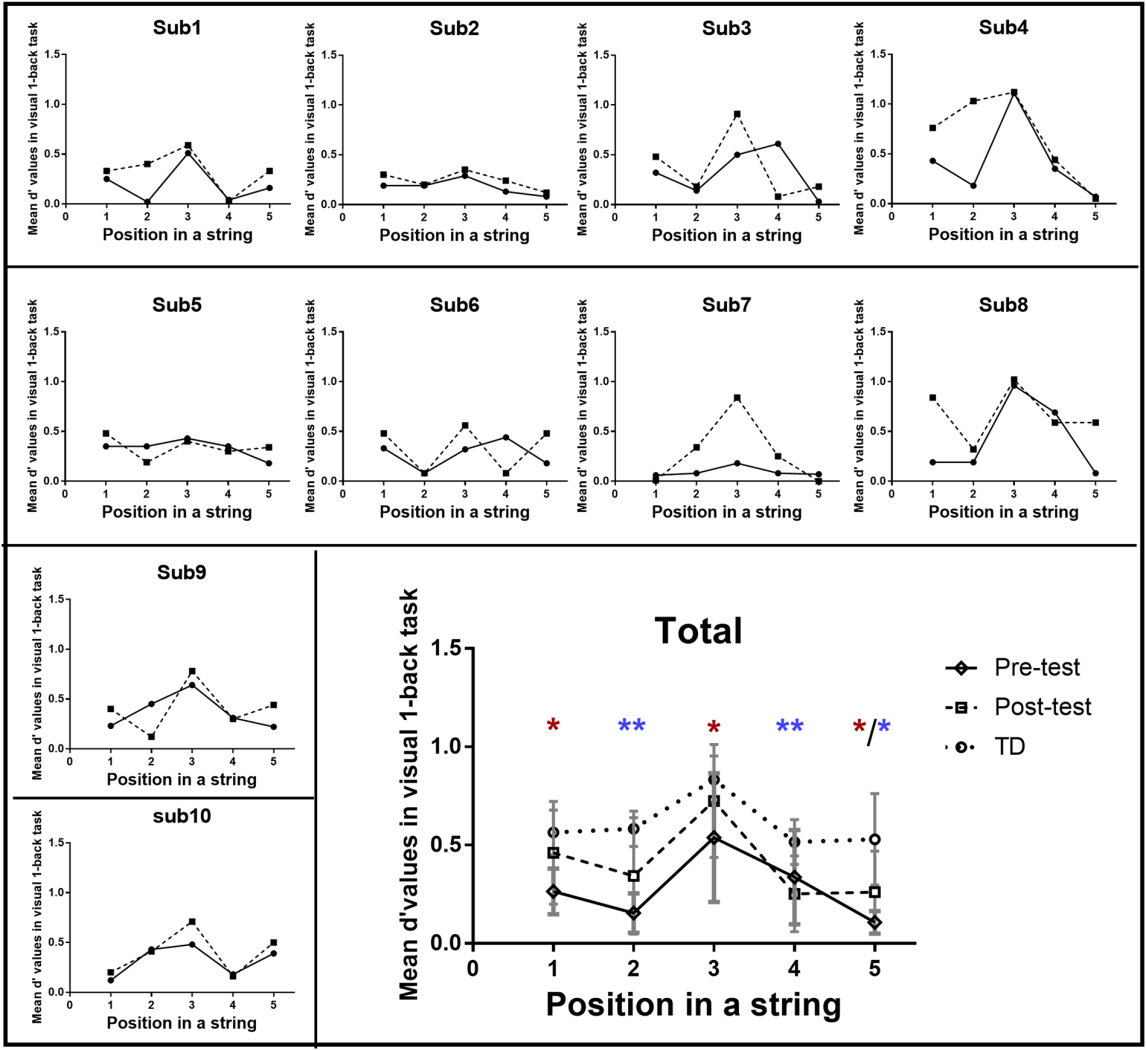
Individual and group data of the position-based d-prime values in visual 1-back task for the trained dyslexics with visual attention span deficit. Small line charts present the individual data of each participant; the large line chart presents the group average of the position-base d-prime values in the visual task. Error bars in the large line chart indicate standard deviations. Solid lines represent performance before training; dashed lines represent performance after the visual training. Additionally, dot line in the large chart represents the position-based performance in typically developing children. *p < 0.05; **p < 0.01. Red asterisks indicate the significant difference between pre- and post- tests; blue asterisks indicate the significant difference between trained dyslexics with VAS deficit and normal readers in the post-test condition.

The results of a Wilcoxon signed-rank test showed that there was a significant improvement in the mean accuracy between pre- and post-tests in trained VAS-impaired individuals with DD (Z = 2.24, *p* = 0.03) but not in untrained VAS-impaired dyslexic individuals (Z = −1.35, *p* = 0.18), as shown in Fig. 1a. Next, using the Mann-Whitney U test, we found that the performance in the visual 1-back task in trained VAS-impaired dyslexic individuals was worse than that in age-matched normal readers (U = 85.5, *p* = 0.04; Fig. 2a) but similar to that in the untrained group of VAS-impaired dyslexic individuals (U = 30, *p* = 0.83) in the pre-test condition; in contrast, the VAS skills of trained dyslexic individuals did not differ from those of the normal readers (U = 47.5, *p* = 0.56), showing better performance than untrained dyslexic individuals (U = 3.5, *p* = 0.003; Fig. 1a) in the post-test condition. Given the significant improvement only in the trained VAS-impaired individuals with DD, a position-based analysis was further conducted to examine the possible training changes in their visual attentional distribution regarding the VAS. As shown in Fig. 3, the results of Wilcoxon signed-rank tests showed that d-prime values in the visual 1-back task when the target appeared in the 1^st^, 3^rd^, and 5^th^ positions of a string significantly increased after the visual intervention (1^st^ position: Z = 2.38, *p* = 0.02; 3^rd^ position: Z = 2.24, *p* = 0.03; 5^th^ position: Z = 1.96, *p* = 0.05). However, compared to chronological age-matched controls, trained dyslexic individuals with VAS deficits still exhibited lower d’ values in the 2^nd^ (U = 98, *p* = 0.004), 4^th^ (U = 96, *p* = 0.006) and 5^th^ (U = 91.5, *p* = 0.02) positions in the post-test. Referring to previous literature^48^, we further computed individual standard deviations across the five positions in the post-test condition to reflect the inter-position variability across the string and compared the scores between trained VAS-impaired individuals with DD and normal readers to explore their patterns of visual attentional distribution. The results of the Mann-Whitney test showed no significant group difference (U = 38, *p* = 0.24).

For reading measurements, in the pre-test condition, trained VAS-impaired dyslexic individuals performed worse than the age-matched normal readers on single character reading speed in both oral (U = 86, *p* = 0.04) and silent (U = 92, *p* = 0.014) modes, as well as on judgement accuracy in the oral (U = 80.5, *p* = 0.04) and silent (U = 87.5, *p* = 0.008) sentence reading tests (Fig. 2a). Compared to untrained VAS-impaired dyslexic individuals, the reading performance of these trained dyslexic individuals showed no significant differences in the pre-test (*p*s > 0.1). These originally lower reading scores of the trained VAS-impaired dyslexic individuals significantly improved after VAS-related training (as shown in Fig. 1a, by the Wilcoxon signed-rank tests, oral character speed: Z = 2.37, *p* = 0.02; silent character speed: Z = 2.52, *p* = 0.012; oral sentence accuracy: Z = 2.12, *p* = 0.03; silent sentence accuracy: Z = 2.37, *p* = 0.02), their scores in most of the reading tests were similar as those of age-matched normal readers (Fig. 2a, in silent character reading speed: U = 79.5, *p* = 0.11; in judgement accuracy of oral sentence reading: U = 71.5, *p* = 0.16; in judgement accuracy of silent sentence reading: U = 75, *p* = 0.10; but not in oral character reading speed: U = 89.02), revealing that they were close to the normal level. In contrast to the baseline control group of untrained VAS-impaired dyslexic individuals (Fig. 1a), a significant difference between pre- and post-tests was only observed in silent reading speed at the single character level (Z = 2.38, *p* = 0.02). These untrained dyslexic individuals showed lower accuracy (U = 21.5, p = 0.045) and slower responses (U = 27.5, *p* = 0.03) in silent sentence reading than trained VAS-impaired dyslexic individuals in the post-test.

Given that only VAS-impaired dyslexic individuals exhibited training effects on VAS and reading skills, the relationship between the training effects of VAS and reading skills was explored. Based on the abovementioned significant improvements, we computed the differences in the mean accuracy on the visual 1-back task and the d-prime values in the 1^st^, 3^rd^, 5^th^ positions between post- and pre-tests, as well as the changes in reading performance at the character and sentence levels, which underwent Spearman correlational analyses. Relevant results showed that the change in silent sentence reading accuracy was significantly correlated with the subtractions of the mean accuracy (r_spearman_ = 0.71, *p* = 0.04) as well as d-prime values of the 3^rd^ position (r_spearman_ = 0.79, *p* = 0.02) in the visual 1-back task. Moreover, there was no other significant correlation (*p*s > 0.1). A follow-up test was conducted three months after training. The performance improvements in silent sentence reading accuracy and VAS capacity in the trained group of VAS-impaired dyslexic individuals were still apparent, as revealed by non-significant differences in Wilcoxon signed-rank tests between the post-test and the follow-up test (*p*s > 0.1). Especially for the follow-up tests, these trained dyslexics exhibited better performance in the silent sentence reading performance (U = 1.00, *p* = 0.02) and VAS skill (U = 7.50, *p* = 0.008) as compared to the untrained dyslexics with impaired VAS. These untrained dyslexics did not show significant improvements in either silent sentence reading or VAS capacity, while they only showed an increased speed in silent character reading after training. Accordingly, we also examined the stability of the improvements in silent character reading in untrained dyslexic individuals, and the results showed that the reading performance in the follow-up test was worse than that in the post-test (Z = 2.02, *p* = 0.04) but was better than that in the pre-test (Z = 2.03, *p* = 0.04), and this result was probably due to a practice effect.

## Training Effects of VAS and Reading Skills for VAS-normal Individuals with DD

We further analysed the data of trained VAS-normal dyslexic individuals to explore the possible influence of the heterogeneity of DD on the intervention effect. The results of Wilcoxon signed-rank tests (Fig. 1b) showed no significant change in the mean accuracy of the VAS task between the pre- and post-tests in the trained dyslexic children with normal VAS function (Z = −1.12, *p* = 0.26). The VAS capacity of these trained dyslexic individuals was similar to that of age-matched normal readers in the pre- (U = 53, *p* = 0.53) and post- (U = 38.5, *p* = 0.12) conditions (Fig. 2b). Additionally, there was no significant improvement in VAS performance for the baseline control group (i.e., nontrained VAS-normal individuals with DD, Z = −1.36, *p* = 0.17).

These trained VAS-normal dyslexic individuals showed an improvement in their character reading speed in the oral mode (Z = 2.31, *p* = 0.02), which was also observed in untrained VAS-normal dyslexic individuals (Z = 2.32, *p* = 0.02). Compared to the normal level reference group, trained VAS-normal dyslexic individuals performed worse in oral character reading and oral/silent sentence reading in the pre-test condition (oral character: U = 101.5, *p* = 0.02; oral sentence accuracy: U = 87.5, *p* = 0.04; oral sentence speed: U = 94, *p* = 0.02; silent sentence accuracy: U = 87, *p* = 0.04); in addition, these group differences were maintained in the post-test (oral character: U = 100, *p* = 0.02; oral sentence accuracy: U = 71.5, *p* = 0.04; oral sentence speed: U = 84, *p* = 0.002; silent sentence accuracy: U = 72, *p* = 0.04).

Additionally, to directly compare the group differences in the training effects, chi-square tests were conducted to compare the number of participants who exhibited improvements in the VAS or reading performance after training (i.e., post-test scores minus pre-test scores >0) across groups. Fisher’s exact test was adopted to analyse the data. Relevant results showed a significant group difference in the percentage of the improved individuals for both VAS skills [χ^2^ = 16.30, *p* = 0.002] and sentence reading performance [χ^2^ = 11.48, *p* = 0.02]. Further pairwise comparison showed that the trained group of VAS-impaired dyslexic individuals had a higher percentage of individuals with VAS/sentence reading improvements than the other four groups (*p*s < 0.05), and there were no other significant group differences (*p*s > 0.1).

**Figure 4:**
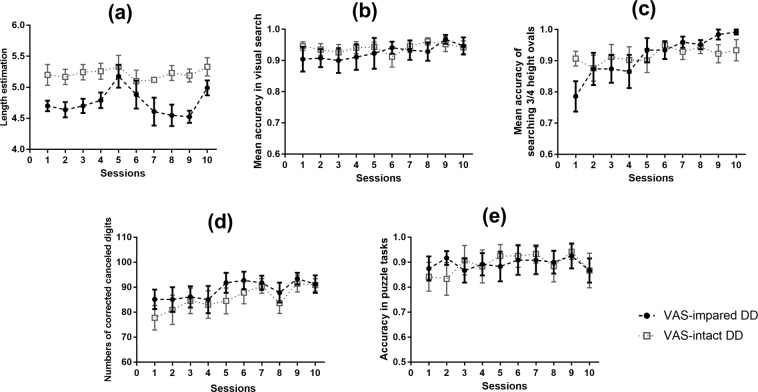
Learning curves for the two groups of trained dyslexics in each of the training task. (**a**), the change of the threshold of length estimation during the 10 sessions. (**b**), the accuracy change in the visual search task during the 10 sessions; (**c**), the change in mean accuracy of searching 3/4 height ovals among circles in the visual search task; (**d)**, the learning curve of subtractions between the total number of digits participants read in the time limitation of 20 seconds and the number of errors; (**e**), the total accuracy in maze and line puzzle tasks. Error bars indicate standard deviations. Dashed lines in black represent performance of VAS-impaired DD; dashed lines in grey represent performance of VAS-intact DD.

The above results revealed that only the trained group of VAS-impaired dyslexic individuals showed a significant training effect on VAS task performance, and similar findings were observed for the learning curve in the 10 training sessions (Fig. 4). VAS-impaired dyslexic children, rather than VAS-normal dyslexic children, exhibited a significant learning effect in training tasks of VAS estimation, visual search, and digital cancelling, and there was no significant change in visual tracking performance that was not included in the following analysis. To explore the possible influence of this visual training on VAS skills, we further conducted Spearman correlation analyses and found that there were close relationships between improvements in the performance of the visual 1-back task (especially the d’ values in the 3^rd^ position of a string) and learning-related changes in the three training tasks of VAS estimation, digital cancelling, and visual search. Detailed results can be found in the Supplementary Materials.

## Discussion

The present study explores the influence of VAS-based intervention on VAS and reading-related skills among Chinese dyslexic children. The results showed that a period of training focusing on two subcomponents of the VAS (i.e., bottom-up attention span and top-down attentional modulation) could lead to enhanced performance in the visual 1-back task, and this improvement was only present in Chinese dyslexic readers with VAS dysfunction and not in the dyslexic children with intact VAS capacity. Specifically, the attentional distribution in VAS-impaired dyslexic individuals was changed following VAS-based training, and increasing sensitivity was observed in the 1^st^, 3^rd^, and 5^th^ positions of a string, further exhibiting similar inter-position variability as that of the age-matched controls. The changes in the mean accuracy and d-prime value of the 3^rd^ position in the visual 1-back task were related to the learning effects during the VAS-related procedure. Moreover, there was significant improvement in sentence reading accuracy, and further correlation analyses showed that improvement in VAS skill and sentence reading accuracy were closely related to each other, suggesting the possible influence of the VAS on sentence reading procedure.

### Visual attention span deficit in Chinese children with dyslexia and its possible influence on their character/sentence reading

In the pre-test condition, the results of group comparisons showed that dyslexic readers exhibited worse performance in the visual 1-back task than chronological-age-matched controls. This finding was consistent with previous results^22^, suggesting VAS impairment in Chinese children with dyslexia. Furthermore, to reduce the influence of the heterogeneity of dyslexia on relevant results and conclusions, we adopted the deviance analysis method to identify dyslexic individuals with a VAS deficit who were invited to participate in the current training study. The proportion of VAS-impaired individuals among dyslexic individuals (i.e., 50%) was larger than that in age-matched normal readers (i.e., 5%). Previous studies have reported that the prevalence of VAS dysfunction in French children with dyslexia is approximately 34%^19^ or 28.1%^18^, while Yeari *et al*.^39^ found no VAS-impaired individuals in a cohort of dyslexic Hebrew adults. The relevant prevalence in the present cohort of Chinese children with dyslexia seemed to be higher than that in dyslexic readers in French or Hebrew. This finding was consistent with the prediction stated in the Introduction section that orthographic depth would modulate the VAS-reading relationship. That is, the VAS may play a more remarkable role in reading in languages with deep orthographies that rely more on a global reading procedure with large units of orthographic input^20,26^. Chinese has a particularly deep orthography^49^, and Chinese reading heavily depends on global processing through the lexical route, especially in the global visual analysis of the form of Chinese characters and the direct mapping between the whole character and relevant semantics, in which the VAS could play an important role. Thus, it could be proposed that the VAS deficit in Chinese dyslexics might be more significant than that in alphabetic languages. Future studies need to directly compare VAS capacity of individuals with dyslexia across different language systems.

Moreover, in the pre-test, the current VAS-impaired dyslexic individuals exhibited worse reading performance at both the single-character and sentence levels than VAS-normal dyslexic individuals and age-matched normal readers, suggesting a relationship between VAS capacity and Chinese reading skills. Although VAS-normal dyslexic individuals showed more reading problems than normal readers, their reading disability might be due to other factors in addition to a VAS deficit, according to the multiple deficit model of dyslexia^2^, such as an impairment in phonological awareness, orthographic processing skills, and morphological analysis, as suggested by the relevant literature^1,50^. However, the VAS is a general cognitive skills, and its impairment would widely and negatively affect language skills requiring VAS skills, thus further hindering reading procedures. Therefore, VAS-impaired dyslexic individuals might exhibit more severe reading problems than VAS-normal dyslexic individuals.

### The effect of VAS-based training on VAS capacity

Following the VAS-related intervention, only trained dyslexic individuals with VAS impairment exhibited significant improvements in their VAS skills. In the pre-test in the current study, VAS-impaired dyslexic readers showed low sensitivity across the five positions of a string in the VAS task; in contrast, in the post-test, their pattern of attentional distribution regarding VAS skills seemed to be normal. In detail, the relevant inter-position variability of these trained individuals with DD was similar to that of the chronological age-matched controls in the post-test, and the homogeneous allocation of their visual attention span resources revealed the pattern of a wide VAS window. Since the participants were required to focus on the centre fixation in the present study, a gradient for attentional distribution was observed even in the post-test condition of totally homogeneous allocation, in which there was a fixation advantage and a decreasing distribution as the eccentricity increased. The sensitivities in the 1^st^, 3^rd^, and 5^th^ positions of a string were increased for the trained VAS-impaired individuals with DD, revealing a global enhancement of identification at both centre and outside positions within the visual attention span. The present training tasks mainly focused on the two subcomponents of the VAS, that is, bottom-up attention ability of visual short-term memory storage (i.e., length estimation task) and top-down attention modulation and control (i.e., digital cancelling and visual search); the correlational analyses revealed that the more participants improved during the intervention procedure, the larger their training effects in visual 1-back task were. Therefore, it could be proposed that the current intervention might improve VAS skills by broadening the span to store more fast-presented visual stimuli in visual short-term memory and by improving the focusing and orientation of visual spatial attention to normally distribute the visual attention resources and to efficiently prioritize targets over distractors.

In particular, we observed a remarkable improvement in the performance of the 3^rd^ position in the visual 1-back task. Another recent study from our group found that the developmental improvement in VAS capacity was heavily reflected by the performance in Position 3 of the string in the visual 1-back task, exhibiting an increasing fixation-position advantage as age increased^51^. This finding was consistent with the abovementioned results in the current training study, suggesting that one of the critical indexes of VAS level was the fixation-position performance in the visual 1-back task. The current training tasks of bottom-up and top-down visual attention exert prior influences on performance at the fixation position in the VAS task. We proposed that if the participants could automatically identify the target in the fixation position after more sufficient attentional training, then this automatization might decrease the requirement of visual attentional processing by saving more attentional resources to be used to process targets at other positions of the string. Of course, this inference needs to be further examined. Moreover, trained VAS-impaired dyslexic individuals still exhibited lower sensitivity in the 2^nd^ and 4^th^ positions of a string compared to the chronological age-matched controls. Since targets in the 2^nd^ and 4^th^ positions of a string were flanked by neighbouring stimuli, the influence of crowding effect would then influence the allocation and distribution of visual attention. In particular, individuals with dyslexia were found to show excessive visual crowding^52,53^. Consequently, surrounding stimuli in the sequence would considerably interfere with the target processing in dyslexic individuals, with further hampering progress in their VAS skills at these flanked positions. Future studies are required to explore whether the discrimination sensitivity in the 2^nd^ and 4^th^ positions would also be improved after a longer period of VAS-based intervention involving the crowding effect more.

### VAS-based training effect improves sentence reading instead of character reading

In the present study, silent sentence reading significantly improved following visual training. Moreover, the improvement in sentence reading accuracy exhibited a close relationship with the training effect of VAS, revealing the possible influence of VAS skills in silent reading processes at the sentence level. It has been suggested that silent reading in Chinese mainly depends on the global mapping between orthography and semantics^54^, and VAS may affect the parallel processing of multiple orthographic units of Chinese characters and the character position coding^55^, which may further be related to the efficiency of sentence comprehension during the silent reading task^45^. Accordingly, an improvement in VAS skill might exert a positive influence on silent sentence reading through its role in the parallel processing of multiple characters’ positions/orders and visual-to-semantic mapping. The present findings support the VAS deficit of dyslexia proposed by Bosse *et al*.^15^ from a direct aspect via the VAS-based training method, and further suggest a possible causal link between the VAS deficit and reading disorders to some extent.

Additionally, we also found an acceleration of reading speed at the single-character and sentence levels following visual training. However, significant improvements were not only found in the trained group of VAS-impaired dyslexic individuals but also in the other groups, such as the nontrained group of VAS-impaired dyslexic individuals and the VAS-normal dyslexic groups, and these training effects were similar across groups. Moreover, the improvements in character and sentence reading speed were not related to the change in VAS-related skills in the trained group of dyslexic individuals. These findings revealed that increasing single-character/sentence reading speed was more likely to reflect the influence of practice effect rather than the contribution of improvement in VAS.

It should be noted that special improvements in sentence reading were mainly reflected in judgement accuracy rather than reading speed. In most previous research^55^, sentence reading fluency was indexed according to the accuracy in the paper-pencil test of the sentence verification task, which could reveal the level of reading accuracy and reading speed at the sentence level in an integrated manner. In the current study, we applied a computerized procedure to examine silent reading speed. However, during silent reading, we cannot ensure that participants read every character in one sentence to comprehend; and the final value was computed as the ratio of sentence length to reading time, which might not accurately measure the reading speed in the silent mode. Future research should find a more reasonable method to directly reflect silent reading speed.

### Possible influence of the setting of the current training procedure

In our study, the trained groups completed ten sessions of VAS-based intervention within four weeks, completing two or three sessions per week. Relevant results revealed that trained dyslexic individuals with VAS impairment exhibited more remarkable improvements in their mean accuracy in the visual 1-back task and sentence reading performance than the other four groups of participants. The total training duration in our study was approximately 5 hours. Although it has been suggested that it is better to conduct 20–30 hours of attentional training to measure remarkable effects^56^, it is possible that a shorter period of attentional training can be effective in children because of their higher level of neural plasticity^57–61^. Previous studies found that short-term training on visual skills could significantly exert a positive influence on participants’ behavioural performance in visual-related tests and brain activity in the dorsal visual stream^62,63^. It has been suggested that level of neural plasticity are higher in developing children than in adults^64^, and participants in the present study were children. Accordingly, it could be inferred that the VAS-based training might exert an influence on relevant brain function in the current participants and further help improve their behavioural outcomes. Future studies should utilize neuroimaging techniques to explore the possible relationship between neural plasticity and the effect of visual training. Moreover, our follow-up study found that the training effects in VAS skills and sentence reading performance for the trained dyslexics with VAS deficit continued after two months, and these improvements were only observed in this trained group but not in the untrained dyslexics. Meanwhile, the comparisons of performance in the follow-up tests showed the trained dyslexics performed better than the untrained dyslexics in the sentence reading and VAS skills. These findings suggested that the improvement in cognitive skills brought about by visual training may be stable.

### The influence of dyslexia heterogeneity on the relevant intervention effect

The two trained groups in the current study exhibited differences in their training effects. Specifically, a significant improvement was observed only in the trained group of VAS-impaired dyslexic individuals, not in the trained group of VAS-normal dyslexic individuals. This finding suggests that the heterogeneity in dyslexia indeed influences the intervention effect. VAS-normal dyslexic children had similar levels of VAS skills as age-matched normal readers. Visual training with a longer duration and higher intensity may be required to remarkably improve VAS skills in VAS-intact dyslexic individuals. Furthermore, group differences in the training effects would benefit from excluding the possibility of the placebo effect. If the attentional and sentence reading improvements were caused by a simple placebo effect and not the specific visual training, then both groups of children might show improvement after training. Future studies should manipulate the difficulty of the VAS task and reset the properties of the training procedure to further explore the underlying mechanism of the possible influence of dyslexia subtypes on the intervention effect.

### Limitations

In the current study, various tasks were used in the intervention procedure. Visual search and digital cancelling tasks were utilized to train the top-down attentional components of VAS skills, and the length estimation task was supposed to involve the identification or categorization of the items that compose the visual array, which was highly associated with bottom-up attentional components of VAS skills; however, it was difficult to know which component in the intervention produced the training effect on the VAS skills. Future studies could adopt neuroimaging techniques to further explore the underlying mechanisms of the positive role of this visual attentional intervention in the training effects on VAS and reading skills. In particular, in the length estimation task in the current training procedure, judging the length of strings did not trigger the identification process. It has been suggested that there is a specific mechanism of length estimation that is distinct from the process of identification^65^. Although the present study found a significant correlation between the learning effect in the length estimation task and the training improvement in VAS capacity, future intervention studies should modify the relevant program to more directly address VAS-related processing. Moreover, improvement due to VAS-related training cannot be separated from the influence of general training effects because there was no VAS-unrelated training group in the present study. Future studies should additionally recruit a training group of dyslexic individuals who received training to employ abilities other than VAS skills (e.g., linguistic skills) to eliminate the influence of a placebo effect. Meanwhile, the sample size in the current study was small, and the present conclusions were mainly based on the results of non-parametrical analyses; thus, more studies are required to arrive at a clear conclusion.

## Conclusions

In summary, the present study found that VAS-based intervention including two subcomponents of the VAS (i.e., the visual short-term memory storage of bottom-up attention and spatial distribution and the inhibition of irrelevant information regarding top-down attentional control) could improve VAS skills in VAS-impaired dyslexic individuals, but not VAS-intact dyslexic individuals, particularly increasing the fixation-position advantage in their visuo-spatial attention distribution. Moreover, improvement in VAS skills could bring about an improvement in silent sentence reading performance, suggesting the possible role of the VAS in reading processes via character position decoding in globally orthographic processing and orthographic-to-semantic mapping. The current results support the VAS deficit hypothesis in the context of Chinese language and suggest basic cognitive skills (i.e., the VAS) as possible markers for identifying children at risk of dyslexia and for instruction promoting fluent reading skills. At the same time, the current findings emphasize the importance of the influence of heterogeneity in the dyslexic population on the intervention effect.

## Methods

### Participants

Twenty dyslexic children with VAS deficits (6 girls), twenty dyslexic children with normal VAS function (5 girls), and fourteen chronological-age-matched typically developing children (7 girls) participated in the present study and were selected from the 4^th^ to 6^th^ grades of one primary school. Both types of dyslexic children were randomly and equally divided into trained and nontrained groups. We began by adopting a standardized vocabulary test and a nonverbal intelligence test to screen children with dyslexia, as these tests are commonly used to identify dyslexic children in Mainland China^22,66–68^; in addition, performance in a task requiring the rapid naming of digits, which is a strong predictor of reading ability^28^; character and sentence reading test scores in oral and silent modes; and the evaluation results of language learning difficulty from relevant Chinese teachers were combined to ensure the validity of the dyslexic screening. Then, a visual 1-back task was applied to further identify dyslexic individuals with VAS impairment. Relevant details of the psychometric screening tests and screening procedures are described below. All participants had normal or corrected-to-normal vision without neurological or ophthalmological abnormalities, and were right-handed that was as judged by the Handedness Inventory (Department of Neurology, Beijing Medical University Hospital). The sample included no participants with ADHD, as judged by the Chinese Classification of Mental Disorder 3 (CCMD-3). Written informed consent was gotten from children’s parents and teachers before the assessment. The study was carried out in line with the relevant guidelines and regulations. The research project was approved by the Research Ethics Committee of the School of Psychology, Capital Normal University.

### Psychometric tasks and procedures administered to identify dyslexic individuals

To screen children with dyslexia from a population of primary school students, we adopted a standardized vocabulary test and a nonverbal intelligence test, which are consistent with other studies on dyslexia in Mainland China^22,66–68^.

#### Raven’s standard progressive matrices (RSPM)

RSPM is a standardized test of nonverbal intelligence with five sets of testing items, with difficulty increasing as the test progresses. For each item, children were shown an incomplete matrix and were required to complete the matrix by selecting the target from six to eight options^69^. There were 60 items in total, participants were required to respond in a time limitation of 40 minutes. The raw score was the number of correct response, which was converted to the standardized score referring to the Chinese norms established by Zhang and Wang^69^.

#### Standardized written vocabulary test

This Chinese character recognition test was used to measure participants’ reading skills. In this test, participants are required to write down compound words with the target morphemes provided on the sheet within a time limitation of 40 minutes^22,70^. For example, the target Chinese character might be “清” (pronounced/qing1/, meaning “clear”; the number represents tone); participants were required to write a new morpheme beside the target to generate a real two-character word. Characters are divided into 10 groups that are sorted by testing difficulty from low to high. The testing difficulty is mainly reflected in the visual complexity and frequency of characters (e.g., characters with a high level of testing difficulty would have complex visual forms and low character frequency). The total number of characters in this vocabulary test was 207 for 4^th^-graders, 174 for 5^th^-graders, and 210 for 6^th^-graders, and it was normal that some students could not complete the whole test within the time limitations. A child responded correctly if he/she wrote, for example, “清水” (/qing1 shui3/, which means “clear water”) or wrote another proper word that includes the target morpheme “清”. Each correct response awarded one point. The score for each item group was calculated by multiplying the total points by the relevant coefficient of difficulty. The relevant coefficient of each subgroup across grades was attached in Table 1. The final score for each participant was the sum of sub-scores for all ten groups, which was regarded as an estimation of the child’s vocabulary size.

**Table 1:** The coefficients of each subgroup for the vocabulary test and the relevant formula across grades.

Grades	C1	C2	C3	C4	C5	C6	C7	C8	C9	C10	Constant
Grade 2	12.12	6.89	5.50	4.71	4.63	4.67	5.10	5.59	6.63	8.70	—
Grade 3	17.2	10.15	8.56	7.72	7.21	7.46	7.92	8.14	10.33	14	—
Grade 4	17	11.38	9.30	8.59	8.43	8.47	8.78	9.65	11.73	16.20	449
Grade 5	17.39	12.45	10.2	9.06	9.17	9.18	9.33	10.25	12.41	16.67	989
Grade 6	16.29	11.64	9.50	8.54	8.21	8.10	8.32	9.58	11.71	16.67	1305
Formula	Estimation of vocabulary size = C1*a1+ C2*a2+ C3*a3+ C4*a4+ C5*a5+ C6*a6+ C7*a7+ C8*a8+ C9*a9+C10*a10+Constant size = C1*a1 + C2*a2 + C3*a3 + C4*a4 + C5*a5 + C6*a6 + C7*a7 + C8*a8 + C9*a9 + C10*a10 + Constant										

Note. C1–C10 represent relevant coefficient in each condition; a1–a10 mean the number of correct response in each subgroup.

#### Screening procedure of dyslexia

Referring to relevant literature^22,66–68^, the inclusion criteria adopted to screen the dyslexic individuals from the current pool of Chinese children were as follows. (1) The nonverbal intelligence was in the normal range. As indicated by Zhang *et al*.^69^, if the test score of one participant was higher than the value in the 5th percentile of the norm, then this participant was regarded as having normal nonverbal intelligence. (2) The score on the vocabulary test was at least −1.5 standard deviations below the average of the same-grade children. In this manner, 55 children with a risk of dyslexia were selected from 1048 primary school students (prevalence of 5.25%). Moreover, we invited Chinese teachers to evaluate whether the screened individuals exhibited any difficulty in their daily Chinese learning, and six of the screened individuals were regarded as performing normally or learning Chinese well, and the teachers thought that their low scores in the vocabulary test probably were due to the intentionally unfinished testing. For the remaining 49 participants, we further invited relevant classroom teachers to judge whether a participant had ADHD with the Chinese Classification of Mental Disorder 3 (CCMD-3), and eight students were discarded because they had a high possibility of having ADHD. Moreover, to control for the possible influence of handedness, we excluded another participant because of his left handedness. Finally, the remaining 40 dyslexic readers participated in the following study. Forty normal readers of the same chronological age were selected as a cohort of age-matched controls; these individuals would be the references to help identify VAS-impaired dyslexic individuals. This point will be elaborated later. To further ensure the screening validity of the dyslexia diagnosis, we also compared performance in tasks requiring rapid naming, oral/silent character reading and oral/silent sentence reading between dyslexic and normal readers, and the details of these reading tests are shown below. The results of independent t-tests showed that dyslexic children exhibited lower scores in most of the reading-related tests than normal controls, revealing reading problems at basic, single-character and sentence levels in the current dyslexic individuals (Table 2).

**Table 2:** Descriptive statistics of background information and reading-related skills for each groups.

Items	DD			TD ③ (N = 40)	T test (Total DD vs. TD)	F test (comparison across group ①②③)	Comparisons
	Total DDs	VAS-im DDs ① (N = 20)	VAS-no DDs ② (N = 20)				
Chronological age *(years)*	10.06 (1.50)	10.10 (0.35)	10.03 (0.34)	10.09 (0.39)	—	0.02	—
Gender	29 boys/11 girls	14 boys/6 girls	15 boys/5 girls	19 boys/21 girls	—	—	—
Vocabulary	1477 (161)	1463 (162)	1492 (157)	2328 (179)	3.56**	8.07***	➀➁ < ➂
Non-verbal IQ	36.30 (6.76)	36.31 (1.48)	38.18 (1.43)	37.31 (1.64)	—	1.89	—
RAN (seconds)	13.98 (3.22)	14.32 (0.76)	13.88 (0.74)	10.14 (0.84)	−2.35*	3.93*	➀➁ < ➂
Char_O *(c/min)*	84.03 (25.26)	84.13 (6.24)	83.94 (6.05)	111.23 (6.92)	3.21**	5.55**	➀➁ < ➂
Char_S *(c/min)*	114.90 (37.18)	95.13 (7.99)	133.53 (7.75)	143.77 (8.86)	2.20*	9.76***	➀ < ➁➂
Sen_O_acc *(%)*	0.75 (0.08)	0.74 (0.02)	0.76 (0.02)	0.84 (0.02)	3.36**	6.02**	➀➁ < ➂
Sen_O_spe *(c/min)*	131.82 (62.18)	137.96 (16.55)	126.05 (16.06)	180.97 (18.36)	2.29*	2.70^+^	➀➁ < ➂
Sen_S_acc *(%)*	0.73 (0.11)	0.70 (0.03)	0.76 (0.03)	0.84 (0.03)	3.29**	7.30**	➀ < ➁ < ➂
Sen_S_spe *(c/min)*	273.73 (114.74)	288.68 (36.98)	259.66 (35.87)	340.68 (41.02)	—	1.11	—

Note. DD, dyslexia; TD, age-matched normal readers. VAS-im DDs, dyslexics with impaired visual attention span; VAS-no DDs, dyslexics with normal function in visual attention span. Non-verbal IQ, raw scores in RAVEN test; RAN, naming speed in digit rapid naming task; Char_O, character reading speed in the oral mode; Char_S, character reading speed in the silent mode. Sen_O_acc, judgement accuracy in the oral sentence reading task; Sen_O_spe, oral reading speed in the sentence reading task. Sen_S_acc, judgement accuracy in the silent sentence reading task; Sen_S_spe, silent reading speed in the sentence reading task. *p < 0.05; **p < 0.01.

### Task and procedure to identify individuals with VAS deficits

#### Visual 1-back task

**Figure 5:**
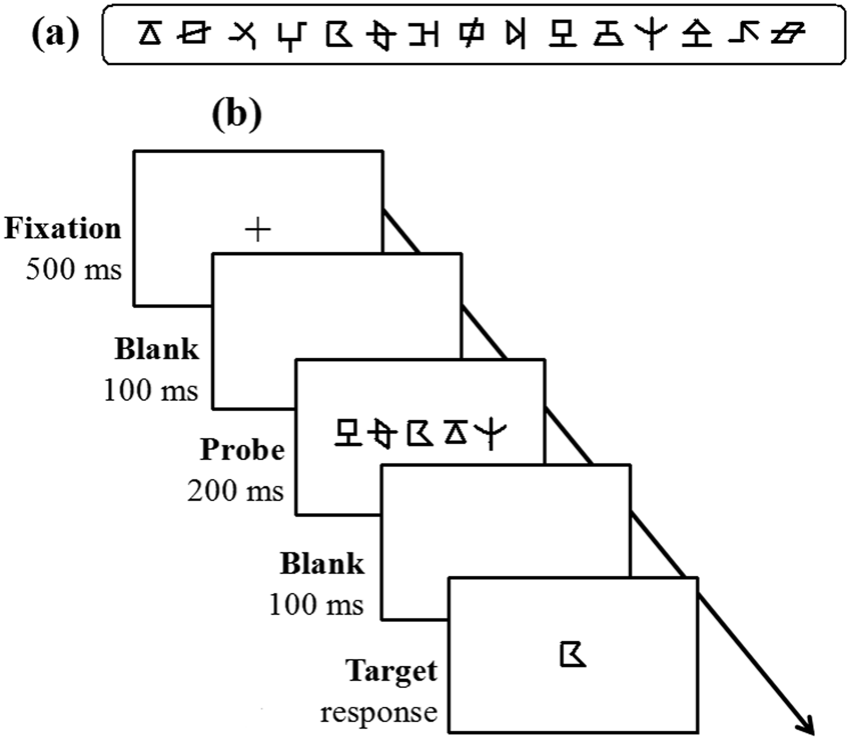
The presentation format of each trial in the visual 1-back task. (**a**) shows all the 15 figures used in the visual 1-back task. (**b**) displays the procedure in visual 1-back task. In each trial, a 500-ms fixation point was first presented at the screen centre, followed by a 100-ms blank and then a 200-ms probe of the five-figure string centred on the fixation. The string was followed by a 100-ms blank, and finally, a target of a single figure appeared below or above (each for half of the trials) the median horizontal line. Participants were required to press different keys to judge whether the target figure was present in the above string or not.

Consistent with relevant studies^22,26,51^, the visual 1-back task was adopted here to measure VAS skills with nonverbal stimuli and nonverbal responses in pre- and post-tests. The test-retest reliability was 0.81. The stimuli in this test consisted of 15 figures (Fig. 5a). The visual complexity of the 15 figures was assessed by 20 undergraduates (12 female) who did not participate in the formal experiment. We used a five-point rating scale during the evaluation, in which 1 point represents “The figure is extremely simple” and 5 points represent “The figure is extremely complex”. Results showed that the average of visual complexity of these figures was 2.27 ± 0.05, revealing low to mid-level degrees of complexity. The visual complexities of any two of the 15 figure did not significantly differ [F(14, 266) = 0.48, *p* = 0.81, *η*^2^ = 0.05]. A list of 80 five-figure strings was created with the 15 figures, and no string included the same figure twice. The visual angle of the string was 7.9° × 0.8° at a viewing distance of 50 cm, with the centre-to-centre distance between each neighbouring figure being 1.7°. These visual stimuli were presented in black on a white screen on a Dell laptop. The display resolution was set at 1024 × 768 with a monitor refresh rate of 75 Hz. The presentation format of each trial was as below: a 500-ms centrally-presented fixation, a 100-ms blank screen, a 200-ms centrally-presented probe of a five-figure string, a post-mask of a 100-ms blank screen, and finally a target of a single figure below or above (half of the trials) the median horizontal line (Fig. 4b). This procedure was in line with that of relevant literature^22,26,51^. Participants were required to judge whether the target was present or absent in the probe string just appearing by pressing corresponding keys as quickly and accurately as possible, with pressing the Z key in the target-present condition and to press the B key in the target-absent condition. After response, another blank screen was present for a random interval (changing from 1000 ms to 1500 ms) between successive trials. Eighty trials including 40 target-present trials and 40 target-absent trials were presented in a random order. The test trials were preceded by 10 practice trials. This test was programmed by Eprime 1.1, and the response time and accuracy were recorded. The reaction time in the present study might involve visual coding of the target figure, search and retrieval from the relevant resources in short-term memory, decision-making, selections between different keys, and motor action of pressing keys. In contrast, the data relating to accuracy in the visual 1-back task might be more appropriate to reflect the VAS capacity. Accordingly, the following analyses were mainly conducted to determine the mean accuracy of the visual task. Moreover, we further computed d-prime values based on the accuracy of each position of a string for further position-based analysis.

#### Identification procedure of individuals with a VAS deficit

A deviance analysis method^71^ was adopted to select the individuals with a VAS deficit. According to this method, the dataset of normal readers with extremely high (3 standard deviations) or low (−3 standard deviations) accuracy in the visual 1-back task were first excluded, and two datasets of normal readers were eliminated because their accuracies were higher than 3 standard deviations of the group average; second, we computed the one-tail 95% confidence limit of the remaining 38 normal readers’ accuracies in the VAS task (i.e., −1.65 standard deviations) as the criterion for classifying the dyslexic children with VAS dysfunction, and the cut-off value was 0.55 for the mean accuracy in the visual 1-back task. Individuals with an accuracy lower than the cut-off value were classified as having a VAS deficit. Finally, twenty of the 40 children with dyslexia (50%) were identified as having VAS dysfunction, while two of the 38 age-matched normal readers (5.26%) exhibited a VAS deficit (Table 2). In the present training study, the 20 VAS-impaired and 20 VAS-intact dyslexic children were randomly and equally divided into trained and nontrained subgroups (Table 3). Fifteen normal readers were randomly selected from the 36 controls with intact VAS function (i.e., the other two normal readers with VAS impairment were excluded) and were recruited for the current intervention study. In addition, one of the normal readers was excluded from the final data analysis because his data were unintentionally lost in the post-test condition.

**Table 3:** Group comparisons between non-trained and trained dyslexics in each condition.

Items	Trained VAS-im DDs (N = 10)	NonTrained VAS-im DDs (N = 10)	Mann-Whitney U (Asymp. Sig. p value)	Trained VAS-no DDs (N = 10)	NonTrained VAS-no DDs (N = 10)	Mann-Whitney U (Asymp. Sig. p value)
Chronological age *(years)*	9.64 (1.03)	10.58 (0.90)	43.5 (0.23)	9.85 (1.55)	10.75 (0.79)	54 (0.10)
Gender	7 boys/3 girls	7 boys/3 girls	—	8 boys/2 girls	7 boys/3 girls	—
Vocabulary	1441 (348)	1485 (362)	38 (0.53)	1477 (637)	1813 (493)	53 (0.11)
Non-verbal IQ	37.02 (8.38)	35.63 (6.39)	39.5 (0.43)	38.29 (6.10)	37.38 (6.21)	26 (0.33)
RAN (seconds)	15.63 (4.54)	13.01 (2.18)	20 (0.21)	12.68 (3.85)	13.03 (1.50)	49 (0.21)
Char_O *(c/min)*	86.63 (34.83)	81.63 (32.55)	33.5 (0.88)	84.29 (18.49)	84.50 (14.82)	33.5 (0.81)
Char_S *(c/min)*	94.63 (35.03)	94.63 (23.60)	33 (0.92)	136.29 (40.21)	137.25 (34.86)	44.5 (0.41)
Sen_O_acc *(%)*	0.75 (0.08)	0.73 (0.06)	25 (0.46)	0.76 (0.11)	0.79 (0.04)	34.5 (0.44)
Sen_O_spe *(c/min)*	142.09 (81.27)	129.83 (47.44)	30 (0.83)	128.72 (39.14)	134.84 (50.62)	30 (0.82)
Sen_S_acc *(%)*	0.70 (0.11)	0.69 (0.13)	29.5 (0.79)	0.77 (0.12)	0.77 (0.10)	27.5 (0.95)
Sen_S_spe *(c/min)*	307.02 (82.18)	270.34 (90.03)	26 (0.53)	260.34 (115.41)	298.52 (108.64)	32 (0.64)
VAS ACC (%)	0.52 (0.03)	0.52 (0.02)	30 (0.83)	0.61 (0.03)	0.62 (0.03)	36.5 (0.96)
VAS d-prime	0.27 (0.32)	0.32 (0.52)	45 (0.17)	0.60 (0.24)	0.51 (0.40)	23 (0.21)

Note. Trained VAS-im DDs, trained dyslexics with impaired visual attention span; NonTrained VAS-im DDs, nontrained dyslexics with impaired visual attention span; Trained VAS-no DDs, trained dyslexics with normal function in visual attention span; NonTrained VAS-no DDs, nontrained dyslexics with normal function in visual attention span. Non-verbal IQ, raw scores in RAVEN test; RAN, naming speed in digit rapid naming task; Char_O, character reading speed in the oral mode; Char_S, character reading speed in the silent mode. Sen_O_acc, judgement accuracy in the oral sentence reading task; Sen_O_spe, oral reading speed in the sentence reading task. Sen_S_acc, judgement accuracy in the silent sentence reading task; Sen_S_spe, silent reading speed in the sentence reading task. VAS ACC, mean accuracy in the visual 1-back task; VAS d-prime, mean d’ values in the visual 1-back task.

### Reading-related tasks

Reading-related skills in the present study were measured by a rapid naming task, single-character reading tasks, and sentence reading tasks. In detail, the rapid naming task was only tested in the pre-test condition to examine the screening validity of the dyslexic individuals considering that the rapid naming skill was a significant predictor of reading ability^33^. Single-character and sentence reading tasks in oral and silent modes were conducted in both pre- and post-tests to explore the training-related transfer effect on reading performance.

#### Rapid naming task

Consistent with previous research^51,72^, a rapid naming task of digits was utilized. Two digital matrices were generated with Arabic digits 2, 4, 6, 7, and 9 presented in a random order. Each matrix contained 6 rows, with 5 digits in each row. The two matrices of the digits were printed in 36-point, Times New Roman font on two A4 sheets. Participants were asked to read each of the digit matrices as quickly and accurately as possible from left to right, row by row. For each reading, the completion time was recorded by a stopwatch, and we calculated the average time as the final score of the test.

#### Single-character reading test

This test was a character-list reading task with a time limit of 1 minute, separately performed in silent and oral modes. Because the silent and oral modes used the same reading materials, character reading tests of these two modes were separated by the visual attention test to reduce the practice effect. The split-half reliability was 0.93^45^. Children were presented with a list of 400 Chinese characters intermixed with 13 noncharacters. The main task for participants was to silently/orally read Chinese characters as quickly and accurately as possible within the time limit while occasionally crossing out the noncharacters. The noncharacters were printed in the same font and size as the real characters but could be easily identified as a “noncharacter” by participants. The purpose of using identifiable noncharacter stimuli was to assure participants truly read each character, especially in silent mode. At the end of this test, participants were asked to mark the last item they read. Consistent with relevant research^49^, the score was computed as the number of items read minus the number of errors, in which errors included non-identified noncharacters and incorrectly-marked real-characters. The score was the number of characters read in one minute (character/minute, c/min).

#### Sentence reading test

A sentence verification task was utilized to measure sentence reading performance^45,49,51^. The split-half reliability was 0.85. Similar to other reading fluency tests in Chinese^73^, the sentences consisted of real characters with the number of characters in each sentence varying from seven to twenty-two in the present study. The task included 54 obviously true or false sentences, in which 4 sentences were in the practice session and 50 sentences in the formal experiment (25 sentences were true, and the other 25 sentences were false). All of the characters in sentences were of high frequency. The grammatical structure of all the sentences was “subject + verb + object[+prepositional phrase in some of the sentences]”, such as “一年有十三个月”, meaning “There are thirteen months in a year” (this sentence is false). Previous studies usually used a paper-by-pencil test to measure sentence reading fluency^47,73^, in which participants needed to silently read and judge the correctness of each of the sentences in a list within a time limit (e.g., 3 minutes). The final scores of the test included the number of correctly-judged sentences or the total number of characters in the corrected marked sentences. However, this task included not only fluent reading but also judgement making. Consistent with previous studies^45,51^, the current study thus applied a computerized test of reading fluency, in which the reading fluency procedure and correctness judgements were separate from each other. Accordingly, the relevant indexes of this reading fluency test included speed for the fluent reading procedure and judgement accuracy, similar to previous studies. Referring to relevant literature^51^, the details of each trial in the sentence reading test were as follows: a 500-ms centrally-presented fixation cross was first presented, which was followed by the complete appearance of a sentence that was one line long. Participants needed to read the sentence aloud or silently as accurately and quickly as possible and to press the space bar once they had finished reading this sentence. The reading duration was recorded as the interval between the onset of the sentence the time of pressing the space bar. We could then compute the sentence reading speed as the ratio between the number of characters in one sentence and the corresponding reading duration, and the unit of the reading speed was also converted to the number of characters read in one minute (c/min). After pressing the space bar, participants were asked to judge the correctness of the sentence by pressing the “f” key for true sentences and the “j” key for false sentences. The accuracy of the correctness judgement was also recorded. This test was programmed by Eprime 1.1 in a Dell laptop, and the reading speed and judgement accuracy were further analysed.

### Training tasks

It has been suggested that a reduced visual attention span might potentially arise from different types of underlying mechanisms^74^, including bottom-up stimulus-driven attention abilities (e.g., a limitation on the maximum number of elements that can be stored in one’s visual short-term memory) and top-down controlled attention (e.g., the spatial distribution of attention and efficiency in prioritizing targets over distractors). Accordingly, we utilized training tasks involving the two abovementioned types of attentional capacities. Namely, a length estimation task was applied to train the visual short-term memory storage to address bottom-up attention, and visual search and digit cancelling tasks were used to target the top-down attentional modulation and control. In addition, given that eye movements during VAS tasks might exert an influence on the final performance^37^, the present intervention also included visual tracking tasks to train eye-movement control. Detailed information on each training task is included below.

#### Visual short-term memory storage driven by bottom-up attention

**Figure 6:**
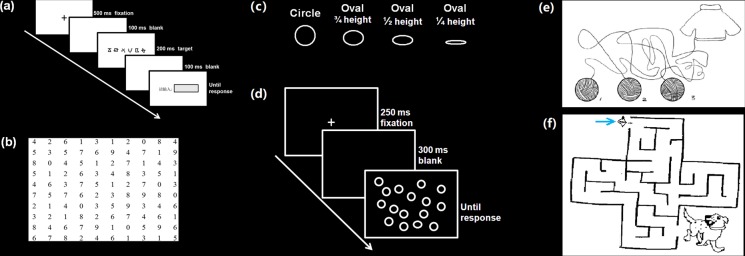
Visual tasks and stimulus materials in the training procedure. (**a**) shows the presentation format of each trial in the estimation task of visual attention span. (**b**) shows the presentation form of digit canceling task. (**c**) displays the circle and oval stimuli in the training task of visual search. (**d**) shows the presentation format of each trial in visual search task. Participants were required to search for an oval among the circles by clicking the left button of the mouse at the corresponding location, and the height of the target oval equally and randomly changed in three conditions. (**e**) is an example in the training task of line puzzle, in which participants were required to find which wool ball is linked to the sweater. (**f**) is an example for the training task of maze puzzle, and the participants should find a proper way from the enter marked by the arrow to the exit next to the dog, and then to draw the route by a pen or pencil.

A length estimation task using the constant stimuli method was designed to train the largest amount of distinct visual elements that could be parallelly processed in a multielement array. The stimuli were the same 15 figures used in the visual 1-back task in the pre- and post-tests. A list of 130 figure strings was created using the 15 figures (10 strings for the practice session and the remaining 120 strings for the formal task). No string included the same figure twice. The horizontal length of the string ranged from 4 to 15 figures, with 10 trials for each length condition. The visual angle of each figure was 0.8° × 0.8°, and the centre-to-centre distance between each adjacent figure was 1.7° at a viewing distance of 50 cm. This test was programmed using E-Prime 1.1 on a Dell laptop and presented in black on a white screen. The display resolution was set at 1024 × 768 with a monitor refresh rate of 75 Hz. Each trial followed the presentation format as below (Fig. 6a): a 500-ms fixation cross, a 100-ms pre-mask of a blank screen, a 200-ms string, a 100-ms post-mask of a blank screen. Participants were then required to input the number of figures in the above string with the keyboard. Following the number input, participants were required to press the ENTER key to continue the task program. A blank screen was presented between successive trials, with a random interval (from 1000 ms to 1500 ms). Finally, the length estimation of the visual array was calculated using the linear interpolation method, which corresponded with an accuracy of 75% to judge the length of a string correctly.

#### Top-down attentional modulation and control

Digit cancelling and visual search tasks were used to target top-down attention related to visual attention span.

The *digit cancelling task* was a paper-and-pencil test^67^ in which participants were required to search for a certain digit (a random integer between 0 and 9) rapidly in a 100-digit matrix (10 across by 10 down) within a time limit of 20 s (Fig. 6b). Once the specific digit was found, participants were required to delete each of them by drawing “\”. At the end of one test, participants were asked to mark the final digit they read. The total number of digits they read within the time limit and the number of errors they performed when deleting digits were recorded, and the score was the total number of digits the participants read minus the number of errors. Every participant was tested twice, and the specific digits that participants were supposed to seek in the two tests were different from each other. The final score was the average of the two tests.

*The visual search task* is similar to the one in the study by Lin *et al*.^75^. The stimuli were circles and ovals (Fig. 6c). The visual angle of each circle was 1.5° × 1.5° at a viewing distance of 50 cm. The ovals had the same width as the circle and included three heights: 1/4, 1/2, and 3/4 of the circle’s diameter. The horizontal and vertical angles subtended 1.5° × 0.4°, 1.5° × 0.8°, and 1.5° × 1.1°. Th stimuli were presented in white on a black screen using E-Prime 1.1 on a Dell laptop. The display resolution was set at 1024 × 768 with a monitor refresh rate of 75 Hz. There were 9 practice trials prior to the formal test. The presentation format of each trial was consistent with the study by Lin *et al*.^67^. In each trial (Fig. 6d), a 250-ms fixation cross was first presented in the screen centre, followed by a 300-ms blank screen, which was followed by a matrix of fifteen circles and an oval on the screen. The task was to find the oval among the circles. Participants were asked to hit the oval as quickly and accurately as possible by clicking the left mouse button when they found the oval. The position of the oval was randomly arranged. A fixation appeared at the screen centre for a random interval (from 200 ms to 800 ms) between successive trials. The 72 trials for the formal test were presented in a random order and included 24 trials for each size of the target oval. The accuracy and reaction time were recorded. Due to the complex processes involved in the index of reaction times, we mainly focused on the accuracy of the visual search task in the following analysis. Moreover, it has been suggested that VAS skills were related to visual search loading more visual attentional resources than to visual search in a pop-out condition^76^. Given that the visual search training task in the present study adopted three types of target ovals, with the same width as the circle and three heights (i.e., 1/4, 1/2, and 3/4 of the circle’s diameter), and identifying the target oval with 3/4 height of the circle’s diameter from the background circles required substantial involvement of visual attention due to the high similarity between this type of target oval and background circles. Therefore, in addition to examining the mean performance in the visual search, we also explored the learning effect in the 3/4 height condition within the two trained groups.

#### Eye-movement control

Paper-and-pencil puzzle tasks (including a line puzzle and maze puzzle) were used. In the line puzzle task (Fig. 6e), participants were required to follow the correct line to find the correct object in connection with the target. Each correct connection was worth one point, and the total score was eight. In the maze puzzle task (Fig. 6f), participants were required to find the correct path from entrance to exit. This task included four items, and each correct response was worth one point. The stimuli were different in each training session. The mean accuracy was computed to be the final score.

### Procedure

There are four stages in the present study, including a pre-test, a VAS training period, a post-test, and a follow-up test. The pre-test and post-test were conducted within two weeks before and after training, and the follow-up test was conducted three months after the VAS intervention to explore whether the training effect was long-lasting. The training lasted for 4 weeks and included 10 training sessions (two or three sessions per week), and each session took approximately 30 minutes. During the training period, two training groups of dyslexic children with impaired VAS and normal VAS function received the 10 training sessions, while the two groups of non-trained dyslexic individuals and age-matched normal readers performed free activities. In the pre-, post- and follow-up tests, reading skills and the visual 1-back task were submitted to all groups with similar testing procedures. Participants were tested individually in a quiet room. At the beginning of the experiment, the experimenter explained the procedure in detail from a standard script. According to previous research^51^, oral and silent reading tasks for each level were separated by the VAS test (interval: approximately 15–20 minutes) during the experiment to reduce possible practice effects due to using the same materials for the two reading modes. Therefore, there were three sessions in the pre- and post-tests: the 1^st^ session contained the character reading fluency and sentence reading fluency task in oral or silent modes; the 2^nd^ session was about the VAS task; the 3^rd^ session included the character reading fluency and sentence reading fluency task in the reversed reading mode to that in the 1^st^ session. For example, if character reading fluency in the silent mode and sentence reading fluency in the oral mode were tested in the 1st session, then oral character reading and silent sentence reading were examined in the 3rd session. There was about a 1-min rest between successive sessions. The testing time for one participant was approximately 45–50 minutes in total.

## Supplementary information

Supplementary materials for training procedure and relevant results

## Data Availability

The datasets generated for this study are available on request to the corresponding author.
