# Supplementary material for: Improving sentence reading performance in Chinese children with developmental dyslexia by training based on visual attention span
Source: Sci Rep. 2019 Dec 12;9:18964. doi: 10.1038/s41598-019-55624-7 (PMC6908582; doi:10.1038/s41598-019-55624-7)
Supplement: Supplementary file 1 — Supplementary materials for training procedure and relevant results [file 41598_2019_55624_MOESM1_ESM.pdf]

Improving sentence reading performance in Chinese children with developmental dyslexia by training based on visual attention span

Jing Zhao[1,*], Hanlong Liu[1], Jiaxiao Li[1], Haixia Sun[2], Zhanhong Liu[2], Jing Gao[2], Yuan Liu[2], Chen Huang[1]

[1] Key Laboratory of Learning and Cognition, School of Psychology, Capital Normal University, Beijing, China

[2] Yang Zhen Central Primary School, Beijing, China

*** Correspondence**

Address: Room B902, School of Psychology,

Capital Normal University (East Campus)

23A, Fuwai Baiduizi, Haidian District, Beijing, China

E-mail: conanzj@126.com

Telephone: 86-010-84850571

Supplementary materials for results in the training procedure

Analyses about the learning effects during the VAS-related intervention

The training tasks in the present study involved two types of attentional capacities underlying VAS, that is, bottom-up stimulus-driven attention abilities and top-down controlled attention. In details, a VAS estimation task was applied to train the visual short-term memory storage regarding the bottom-up attention, and visual search and digit cancelling tasks were used to target the top-down attentional modulation and control. In addition, given that eye-movements during VAS tasks might exert an influence on the final performance, the present intervention also included visual tracking tasks to train eye-movement control.

Repeated ANOVAs on training sessions (10 sessions) were separately submitted to the performance in tasks of visual search, digit cancelling, visual attention span, line/maze puzzle tasks within the two groups of training dyslexics. The results of one-sample Kolmogorov-Smirnov tests exhibited no significant effects (*p*s>.1), suggesting normal distribution for all the dependent variables. The results of Levene's test for the homogeneity of variance showed that the variances were equal for all the measures (*p*s>.1). Post hoc analyses were conducted in which the relevant p-values were corrected by Bonferroni adjustment.

Visual attention span estimation

For the training dyslexics with VAS deficit, the training effect was significant [F(9, 63)=2.41, p=.02, η^2=.19], and post hoc analyses showed that the visual attention span in the 5th session was larger than that in the other sessions except for the 10th session (*p*s<.05), and VAS in the 10th session was larger than that in the other sessions except for the 5th and 6th sessions (*p*s<0.05), and there were no any other significant differences (*p*s>0.05).

As to the VAS-normal dyslexics, the training effect was not significant [F(9, 72)=.66, p=.74, η^2=.12].

Digit cancelling

As to the VAS-impaired dyslexics, the training effect was significant [F(9,63)=2.52, p=.013, η^2=.34]. Post hoc analyses showed that participants' performance in Session 6, 7, 9, 10 was better than that in Session 1 to 3 (*p*s<.05), and

accuracy in Session 6, 9, 10 was higher than that in Session 4 (*p*s<.05), meanwhile accuracy in Session 8 was lower than that in Session 9 (*p*=.03).

The training effect of VAS-normal dyslexics was not significant [F(9, 72)=2.68, *p*=.14, η^2=.23].

Visual search

The training effect of the dyslexic children with impaired VAS was significant [F(9, 63)=2.40, *p*=.02, η^2=.23]. Post hoc analyses showed that the accuracy in Session 5 to 10 was higher than that in Session 1 to 3 (*p*s<.05); meanwhile accuracy in Session 9 was higher than that in Session 4 (*p*=.03).

For the VAS-intact dyslexics, the training effect in mean accuracy of visual search was not significant [F(9, 72)=.84, *p*=.48, η^2=.11].

Moreover, it has been suggested that VAS skills were related to visual search loading more visual attention rather than to the visual search in a pop-out condition (Lallier et al., 2013). Given that the training task of visual search in the present study adopted three types of target ovals, which had the same width as the circle and included three heights (i.e. 1/4, 1/2, and 3/4 of the circle's diameter), and identifying the target oval with 3/4 height of the circle's diameter from the background circles required great involvement of visual attention due to the high similarity between this type of target oval and background circle. Therefore, we further explored the learning effect in this condition within the two trained groups. The VAS-impaired dyslexics showed significant differences across sessions [F(9, 63)=2.98, *p*=.009, η^2=.43], in which the accuracy in Session 5 to 10 was higher than that in Session 1 to 4 (*p*s<.05) and mean accuracy in Session 1 was lower than that in Session 2 to 4 (*p*s<.05). As to the VAS-intact dyslexics, there was not a significant effect of session [F(9, 63)=.91, *p*=.52, η^2=.12].

Eye-movement control

As to the total scores in puzzle tasks, the training effect was not significant in either VAS-impaired [F(9,81)=.27, *p*=.98, η^2=.03] or VAS-normal [F(9,81)=1.49, *p*=.24, η^2=.14] dyslexics.

Potential improvement during the VAS-related intervention and its relation to the training effects of VAS skills for the VAS-impaired DDs

The above results revealed that VAS-impaired dyslexic children rather than VAS-normal dyslexic children exhibited the significant learning effect in training tasks of VAS estimation, visual search, and digital cancelling, and there was not significant change in visual tracking performance which was not included into the following analysis. We used slopes of the learning curves in the training tasks as the estimation of their learning effect. Functions of linear, exponent, logarithmic, quadratic and cubic were tried to fit the datasets of all the 10 training sessions for these training tasks except the length estimation task. Especially, performance in the length estimation task reached the highest level in about Session 5/6, seemed to reduce from Sessions 6 to 9, and finally (i.e., in Session 10) returned to a level which did not differ from that of the highest level in Session 5/6. This trend of learning curve might be associated with the fatigue effect during the training procedure. Accordingly, we used the datasets from Session 1 to Session 5 in the length estimation task to conduction the curve fitting. It should be noted that the choice of only using a part of training sessions might lead to inflated positive findings. Future studies with setting a longer period for the training procedure are required to further ensure the learning effect in VAS-related tasks (especially for the length estimation).

As shown in Table S1, the logarithmic function (y = aln(x)+b) was finally selected because it could fit the learning curve of each training task better than the other functions. The "y" included the performance of each session in the three visual training tasks of visual search, digital canceling, and length estimation which all exhibited significant improving trends during the intervention; the "x" represents the session number; the "a" indicates the slope of the learning curve that we would utilize in the following analysis, and b represents the constant.

Table S1 Detailed information in curve fitting procedure

**Function**	**visual search total**		**visual search-3/4**		**digital cancelling**		**length estimation (S1-S5)**		**length estimation (S6-S10)**		**length estimation total**	
	R^2	F value	R^2	F value	R^2	F value	R^2	F value	R^2	F value	R^2	F value
Linear	.52	**8.62***	.74	**22.80*****	.48	5.53+	.72	7.73+	.05	.15	.22	2.20
exponent	.52	**8.79***	.74	**22.70*****	.48	5.57+	.72	7.61+	.05	.16	.22	2.27
logarithmic	.60	**12.02****	.63	**13.33****	.55	**7.47***	.82	**13.33***	.07	.24	.19	1.82
quadratic	.61	**5.49***	.74	**10.01****	.53	2.84	.82	4.56	.05	.05	.26	1.23
cubic	.64	3.54+	.80	8.18*	.67	2.65	.91	3.41	.83	1.58	.32	.96

Note. S1-S10 means Session 1 to Session 10 for the training procedure. Visual search-3/4, performance in searching the oval with a 3/4 height of the circle during the visual search task. +, $p<.1$; *, $p<.05$; **, $p<.01$; ***, $p<.001$.

Given the significant improvements in mean accuracy in the visual 1-back task, as well as the d-prime values when the target was presented in the 1st, 3rd, and 5th positions of a string, we further examined the relationship between the improvement of VAS skills and the learning benefit of the three visual training tasks through Spearman correlation analysis (Table S2). Results showed that there were close relationship between improvements in performance of the visual 1-back task and learning-related changes in the three training tasks of VAS estimation, digital cancelling, and visual search. Especially, the increased d' values in the 3rd position was correlated with learning effects of digital cancelling and length estimation tasks.

Table S2 Correlation coefficients between the improvement of VAS skills and the learning effects of the intervention tasks

	**visual search training**	**visual search training in 3/4 height condition**	**digital cancelling**	**length estimation**
Dif_VASacc	**.99*** **	**.94** **	**.71***	.61
Dif_VASd'1	-.46	-.54	-.24	-.17
Dif_VASd'3	.64	.6	**.93** **	**.83***
Dif_VASd'5	-.09	-.03	.29	.34

Note. Dif_VASacc, the subtraction of mean accuracy in the visual 1-back task between the post- and pre- tests; Dif_VASd'1, the subtraction of d-prime values between the post- and pre- tests when the target was presented in the 1st position of a string in the visual 1-back task; Dif_VASd'3, the subtraction of d-prime values in the 3rd position between the post- and pre- tests; Dif_VASd'5, the subtraction of d-prime values in the 5th position between the post- and pre- tests. *, p<.05; **, p<.01; ***, p<.001.
